# Iron Deficiency and Iron Deficiency Anemia During Pregnancy—Opportunities to Optimize Perinatal Health and Health Equity

**DOI:** 10.1001/jamanetworkopen.2024.29151

**Published:** 2024-08-01

**Authors:** Ashley E. Benson, Jamie O. Lo, Aaron B. Caughey

**Affiliations:** Department of Obstetrics and Gynecology, Oregon Health & Science University, Portland.

Iron deficiency, with or without anemia, affects almost half of pregnancies worldwide, adversely impacting maternal and fetal outcomes. In North America, the estimated prevalence of iron deficiency during pregnancy is reported to be greater than 50%, and nearly 12% of pregnancies are affected by iron deficiency anemia.^1,2^ Iron deficiency anemia is associated with an increased risk of maternal complications, including preterm labor, cesarean delivery, postpartum hemorrhage, and maternal death, as well as fetal complications, like low birth weight and small for gestational age. Iron deficiency anemia is influenced by social determinants of health, such as race, ethnicity, socioeconomic status, nutritional status, and food insecurity. Thus, the US Preventive Services Task Force (USPSTF) examined the evidence regarding the benefits and harms of screening and supplementation for iron deficiency with or without anemia in asymptomatic pregnant individuals^3,4^ to update its previously published recommendations from nearly a decade ago.

In the evidence review and updated recommendation statement,^3,4^ the USPSTF extended its review to include the benefits and harms of screening and supplementation for iron deficiency without anemia on maternal and infant health outcomes in asymptomatic pregnant individuals. Additionally, the evidence review and recommendation statement^3,4^ examined studies on the relationship between change in maternal iron status and improvement in infant and maternal outcomes in pregnant individuals with iron deficiency with or without anemia. After reviewing the literature, the USPSTF did not find sufficient evidence that screening and supplementation for iron deficiency, with or without anemia, during pregnancy improved maternal and infant health outcomes (I statement). Additionally, they found insufficient evidence to determine the balance of benefits and harms of routine iron supplementation in pregnant individuals to prevent adverse maternal and neonatal health outcomes (I statement).^3,4^

As cohort studies suggest an association between latent (or nonanemic) iron deficiency and maternal and neonatal morbidity,^5^ it is timely and relevant that the USPSTF included iron deficiency without anemia in this evidence review.^3,4^ This is a critical step to better understand these associations and the optimal ways to supplement or treat pregnant persons with iron deficiency. However, the USPSTF was unable to find adequate studies to address 4 of 5 key questions, highlighting a significant gap in knowledge. As a result, their ability to make recommendations was inherently limited. The absence of evidence should not be interpreted as evidence of a negative association. Rather, this void emphasizes the value and need for additional studies addressing screening and supplementation.

Importantly, we would like to draw attention to 3 points. First, the insufficient evidence underscored by the USPSTF evidence review and recommendation statement^3,4^ is partly due to the lack of consensus regarding the routine use of screening for iron deficiency in pregnancy and on the screening thresholds for diagnosing iron deficiency. For example, although serum ferritin, a marker of body iron stores, has the highest sensitivity and specificity for diagnosing iron deficiency in pregnancy, there is not a consistently used diagnostic threshold. Ferritin levels of less than 30 ng/mL (to convert to micrograms per liter, multiply by 1) have been endorsed by the American College of Obstetricians and Gynecologists as diagnostic for iron deficiency,^6^ while the World Health Organization has suggested a cutoff of less than 15 ng/mL,^2^ and others have proposed thresholds as high as 50 ng/mL, extrapolated from nonpregnant individuals.^5^ A 2017 systematic review^7^ found that almost two-thirds of studies in pregnant individuals used overly restrictive thresholds of serum ferritin, less than 12 ng/mL to less than 15 ng/mL, for the diagnosis of iron deficiency, largely based on international guidelines informed by consensus meetings undertaken more than 20 years ago rather than evidence-based studies.^7–9^ The discrepancy in ferritin cutoff values used has created ambiguity in interpreting the literature and in identifying the ideal management of iron deficiency during pregnancy, thereby impacting both maternal well-being and neonatal health. Thus, high-quality studies exploring an optimal serum ferritin threshold in pregnancy are critically needed to address this globally prevalent issue.

Second, although data to guide the management of iron deficiency in pregnancy are urgently needed, we emphasize the potential for worsening health disparities with a lack of recommendation for screening or oral iron supplementation in the setting of insufficient evidence. In particular, this may affect Black women in the United States, who experience higher rates of iron deficiency and iron deficiency anemia compared with others.^10^ Research indicates that Black women are less likely to be screened by their health care practitioners for these conditions, resulting in delayed diagnosis and treatment.^11–13^ This gap is troubling, as pregnant Black individuals also face more severe consequences of iron deficiency anemia, such as a 2-fold higher risk of needing blood transfusions^14^ and up to a 5-fold higher risk of death from postpartum hemorrhage compared with pregnant White individuals. These disparities highlight the urgent need to prioritize studies on screening and treatment for those at risk of racial and ethnic inequities.

Third, oral iron is well-established as a safe and effective treatment for iron deficiency and iron deficiency anemia. Because of the lack of studies of treatment for iron-deficiency anemia in a screen-detected population, the USPSTF^3,4^ focused primarily on supplementation doses, with most included studies using doses between 20 and 50 mg of elemental iron. Given the high prevalence of iron deficiency during pregnancy^1,15^ and the increased iron requirements, these doses are likely insufficient for adequate treatment and resulted in the lack of benefit observed. Future studies should evaluate the outcomes of iron supplementation in 3 distinct groups: participants who are iron replete, participants who are iron deficient without anemia, and participants with iron deficiency anemia. We recommend that future guidelines focused on the management of iron deficiency in pregnancy should include literature that differentiates between supplementation for prevention and treatment for diagnosed iron deficiency. In addition, future reviews should distinguish between supplemental and treatment doses of oral and intravenous iron and their impacts on outcomes. Guidance on appropriate supplementation (including formulation, dosage, and timing) is also necessary due to the lack of consensus and the variability in dosing strategies found in the literature.

As the rates of iron deficiency during pregnancy increase, so too may pregnancy morbidity.^16^ Given these significant implications for public health, it is critical to conduct additional studies to examine the advantages and disadvantages of screening for and treating subclinical iron deficiency. To harmonize future study findings, it is important to standardize the diagnostic cutoff values for iron deficiency across different populations and settings and clearly differentiate between indications for supplementation vs treatment. This will also help improve the identification and treatment of at-risk individuals, address disparities in care, and ultimately mitigate adverse pregnancy and offspring outcomes.
